# A Narrative Review of Medication Optimisation in Older Adults with Polypharmacy: From Structured Medication Review to AI-Enabled Medication Safety

**DOI:** 10.3390/healthcare14193240

**Published:** 2026-10-01

**Authors:** Rania Aro, Zyta Beata Wojszel, Pierre Duez, Fabian Lecron, Stéphanie Patris

**Affiliations:** 1 Department for Research Support and Technology Transfer, University of Mons (UMONS), 7000 Mons, Belgium; rania.aro@umons.ac.be; 2 Department of Geriatrics, Medical University of Bialystok, Kilinski Str.1, 15-089 Bialystok, Poland; zyta.wojszel@umb.edu.pl; 3 Unit of Therapeutic Chemistry and Pharmacognosy, Faculty of Medicine, Pharmacy and Biomedical Sciences, University of Mons (UMONS), 7000 Mons, Belgium; pierre.duez@umons.ac.be; 4 Department of Engineering Innovation Management, Faculty of Engineering, University of Mons, 7000 Mons, Belgium; fabian.lecron@umons.ac.be; 5 Unit of Clinical Pharmacy, Faculty of Medicine, Pharmacy and Biomedical Sciences, University of Mons (UMONS), 7000 Mons, Belgium

**Keywords:** polypharmacy, older adults, medication review, deprescribing, potentially inappropriate medications, clinical pharmacy, pharmacovigilance, artificial intelligence, medication safety, medication optimisation

## Abstract

Context: Polypharmacy, commonly defined as the concurrent use of five or more medicines, and excessive polypharmacy, often defined as the use of ten or more medicines, are highly prevalent among older adults with multimorbidity. Although appropriate polypharmacy may be necessary, polypharmacy can be associated with adverse drug reactions, drug–drug interactions, falls, hospitalisation, cognitive impairment, non-adherence, treatment burden and (preventable) harm during transitions of care. Methods: This narrative review examines medication optimisation in older adults, tracing its evolution from structured medication review, pharmacist-led interventions, explicit prescribing and deprescribing criteria to digital decision support, artificial intelligence (AI)-enabled pharmacovigilance and personalised medication-risk management. European implementation perspectives, with Belgium and Poland as illustrative examples, are used to examine how professional roles, reimbursement mechanisms, clinical tools and health-data infrastructure shape medication safety. Results: This review integrates geriatric medication optimisation, European implementation experience and AI-enabled pharmacovigilance within a framework linking risk detection, clinical interpretation and treatment optimisation. Current evidence is more consistent for improvements in prescribing quality and medication-related problems than for hard clinical outcomes such as mortality, falls or hospitalisation. AI-supported approaches may improve risk detection and prioritisation, but their clinical value depends on data quality, interoperability, explainability, external validation, human oversight and prospective evidence of patient benefit. Conclusions: Future geriatric medication safety should combine comprehensive geriatric assessment, explicit clinical criteria, patient-centred deprescribing, pharmacist-led multidisciplinary review, interoperable health data and accountable AI-enabled decision support.

## 1. Introduction

Polypharmacy has become a central challenge in geriatric medicine because population ageing is accompanied by increasing multimorbidity, and the simultaneous application of multiple disease-specific treatment recommendations frequently results in complex medication regimens [1,2,3,4]. Multimorbidity, polypharmacy and frailty are closely interrelated: multimorbidity increases the likelihood of exposure to multiple medicines, while frailty reduces physiological reserve and may increase vulnerability to medication-related harm. Conversely, adverse drug effects, treatment burden and functional decline may further contribute to frailty. Polypharmacy should therefore be interpreted within the broader context of multimorbidity, functional vulnerability and individual treatment goals rather than as an isolated numerical phenomenon.

“*Polypharmacy*” is commonly defined as the concurrent use of five or more medicines, while “*excessive polypharmacy*” is often defined as the use of ten or more medicines [1,2]. These thresholds are useful for epidemiological surveillance and risk stratification but do not determine prescribing quality. Multiple medicines may be appropriate when each treatment has a valid indication, a favourable benefit–risk balance, an acceptable treatment burden, and alignment with patient goals; conversely, a smaller regimen may still contain inappropriate, duplicative, poorly tolerated, or insufficiently monitored treatment [3,4].

The burden of polypharmacy is substantial across Europe but varies according to population, socio-cultural setting and literacy, definitions, prescribing culture, reimbursement, and data source. A 2025 update based on the Survey of Health, Ageing and Retirement in Europe reported that polypharmacy prevalence among older adults varied from 25.0% to 51.8% across participating European countries, increasing from 30.0% among individuals aged 65–74 years to 41.1% among those aged 75 years and older [5]. Such variation likely reflects differences in demographic structure, multimorbidity burden, prescribing culture, access to primary care, reimbursement systems, and the availability of medication-review policies. Administrative and reimbursement datasets may also underestimate total medication exposure because over-the-counter medicines, non-reimbursed products, and dietary supplements are not consistently captured.

National data from Belgium and Poland similarly confirm this particularly high exposure to medications among the most advanced age groups. In Belgium, the 2024 Belgian Health System Performance Assessment [6] reported that 42% of insured persons aged 65 years and older used five or more different medicines in 2022; the prevalence gradually increased with age and reached more than 50% of the insured persons in the 85–89 year age group. In Poland, a retrospective analysis of 2019 National Health Fund dispensation data [7], covering a cohort of approximately 38 million Polish citizens, demonstrated that polypharmacy, defined as “*dispensation of five or more prescription medicines within six months*”, affected 11.6% of the total population, 42.1% of people aged 65 years and older, and 55.0% of those aged 80 years and older. Complementary population-based evidence from the PolSenior project also demonstrated a high prevalence of polypharmacy in older Polish adults and, importantly, included medicines actually used by participants, including over-the-counter products and dietary supplements [8]. Together, these sources illustrate how prevalence estimates depend not only on population characteristics but also on the observation period, operational definition and source of medication data.

The clinical relevance of polypharmacy lies in cumulative medication-related risk rather than medication count alone. Ageing modifies drug exposure and response through reduced renal clearance, altered body composition, changes in hepatic metabolism, increased pharmacodynamic sensitivity, and reduced physiological reserve [9,10,11,12]. These changes interact with multimorbidity, frailty, cognitive impairment, malnutrition, sarcopenia, low health literacy and fragmented care, increasing susceptibility to adverse drug reactions, drug–drug and drug–disease interactions, falls, bleeding, delirium, hypoglycaemia, orthostatic hypotension, cognitive impairment, functional decline, and hospitalisation [4,10,13]. Transitions of care are particularly vulnerable periods because discrepancies between prescribed, dispensed, documented and actually used medicines may lead to therapeutic duplication, unintended discontinuation, inappropriate re-initiation or inadequate monitoring [14,15,16].

Over the past two decades, medication optimisation has evolved from medication-list review towards structured, patient-centred, and multidisciplinary medication management. Explicit prescribing criteria, pharmacist-led reviews, deprescribing frameworks and comprehensive geriatric assessment have strengthened the conceptual and operational basis for addressing inappropriate polypharmacy [17,18,19,20,21,22,23,24]. More recently, digital decision support and artificial intelligence have introduced new possibilities for risk prediction, adverse-event detection, interaction modelling, and active pharmacovigilance [25,26,27,28,29]. More broadly, AI is increasingly being investigated in healthcare for diagnostic support, prognostic modelling and therapeutic decision support; in medication safety, its potential lies particularly in integrating heterogeneous clinical and medication data to identify and prioritise patient-specific risks.

Against this background, this narrative review examines medication optimisation in older adults across the continuum from structured medication review and deprescribing to digital decision support and AI-enabled pharmacovigilance. Particular attention is given to the translation of medication-related risk detection into clinically actionable, patient-centred optimisation.

## 2. Scope and Approach of the Narrative Review

Previous reviews have addressed important components of polypharmacy management separately. Pazan and Wehling synthesised definitions, epidemiology and clinical consequences of polypharmacy in older adults [4], while an updated Cochrane review evaluated interventions aimed at improving appropriate polypharmacy [3]. Other reviews have focused more specifically on deprescribing, medication-review interventions or pharmacist-led strategies [30,31,32]. The present review takes a complementary perspective by integrating geriatric clinical decision-making, medication-review and deprescribing approaches, health-system implementation and emerging AI-enabled medication-safety strategies. The underlying question is therefore not only whether medication-related risk can be identified, but how identified risks can be clinically interpreted, prioritised and translated into meaningful treatment optimisation.

Belgium and Poland were selected as illustrative rather than representative European cases because they provide complementary perspectives on this translation. Belgium has developed community-pharmacy medication-review initiatives, specific screening tools and reimbursement mechanisms, while Poland provides extensive national pharmacoepidemiological data together with population-based geriatric evidence. Examining these contexts side by side allows consideration of how professional roles, reimbursement structures, healthcare organisation and data availability influence the transition from population-level or rule-based risk identification to patient-level intervention. Their complementary strengths and limitations also provide a basis for identifying potential areas for future Belgian–Polish research collaboration in medication safety.

The relevant literature was identified through targeted searches of PubMed/MEDLINE, Scopus, Web of Science and Google Scholar, complemented by citation tracking of key articles and selected grey literature, including reports, regulatory documents and policy documents from the World Health Organisation, European Medicines Agency, Belgian health-system sources, and European Union regulatory frameworks. Searches covered literature available up to August 2026. Search terms were combined across the following concepts: polypharmacy, older adults, multimorbidity, frailty, medication review, potentially inappropriate medication, deprescribing, pharmacist-led interventions, pharmacovigilance, clinical decision support, artificial intelligence, machine learning, natural language processing, Belgium and Poland. Priority was given to systematic reviews, consensus criteria, clinical and implementation studies, pharmacoepidemiological analyses and relevant policy or regulatory documents. Because this was a narrative rather than a systematic review, sources were selected according to their relevance to the thematic objectives of the review rather than through exhaustive systematic screening. No formal risk-of-bias assessment or meta-analysis was performed.

## 3. Clinical Burden and Geriatric Challenges

**Figure 1:**
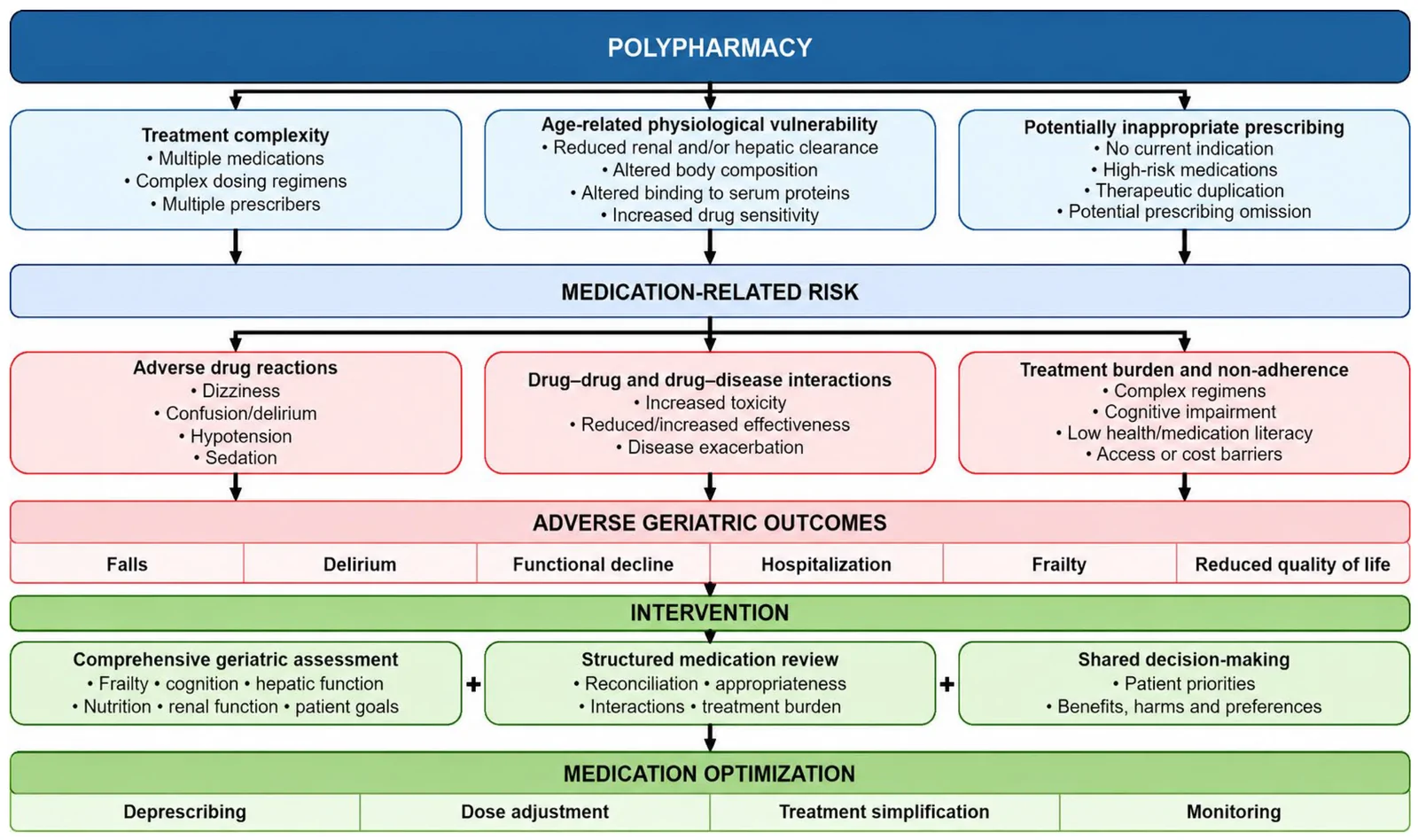
Clinical pathways linking polypharmacy, geriatric vulnerability and medication-related harm in older adults, with potential intervention points for medication optimisation. Colors indicate successive components of the conceptual framework: blue—factors contributing to medication-related risk; red—medication-related adverse effects and geriatric outcomes; green—interventions and medication optimization. Arrows indicate the conceptual direction of the relationships and do not necessarily imply causality.

The clinical consequences of polypharmacy in older adults arise from the interaction between medication exposure, physiological vulnerability, frailty, cognition, functional status and the organisation of care. These risks are heterogeneous and depend on treatment indication, physiological reserve, care setting and patient priorities. Comprehensive geriatric assessment (CGA) provides a useful framework for interpreting medication-related risks in relation to function, cognition, prognosis, life expectancy and individual treatment goals [33,34] (Figure 1).

### 3.1. Adverse Drug Reactions and Drug–Drug Interactions

Adverse drug reactions are among the most clinically significant consequences of polypharmacy in older adults. Their recognition may be difficult because symptoms such as dizziness, delirium, fatigue, falls, urinary retention, anorexia or functional deterioration may be misattributed to ageing or progression of chronic disease. This diagnostic ambiguity contributes to the phenomenon of the prescribing cascade, in which an adverse drug effect is incorrectly interpreted as a new medical condition and results in the prescription of an additional medication [35,36].

Drug–drug interactions may involve pharmacodynamic or pharmacokinetic mechanisms. Pharmacodynamic interactions occur when medicines have additive, synergistic or opposing effects without necessarily altering drug concentrations. Clinically important examples include increased bleeding risk with anticoagulants combined with antiplatelet agents or non-steroidal anti-inflammatory drugs; hyperkalaemia and renal impairment associated with renin–angiotensin system inhibitors combined with potassium-sparing diuretics; excessive sedation from combinations of opioids, benzodiazepines and gabapentinoids; serotonergic toxicity, including serotonin syndrome, associated with combinations of serotonergic medicines such as triptans, monoamine oxidase inhibitors, selective serotonin reuptake inhibitors or amphetamines; and QT prolongation associated with several psychotropic, antiarrhythmic or antimicrobial agents [13,36,37]. Pharmacokinetic interactions occur when one medicine modifies the absorption, distribution, metabolism or elimination of another, thereby altering systemic exposure. These interactions may be particularly important in older adults because age-related changes in renal and hepatic function can amplify the consequences of altered drug clearance [11,12,37].

Drug–disease interactions are equally important. For example, anticholinergic drugs may worsen cognitive impairment or urinary retention; non-steroidal anti-inflammatory drugs may exacerbate heart failure or chronic kidney disease; and sedative-hypnotics may increase the risk of delirium and falls [20].

The clinical significance of these interactions depends on comorbidity, competing indications, physiological reserve and treatment goals. Screening tools can therefore support risk detection but cannot determine medication appropriateness independently of clinical context.

### 3.2. Medication Adherence and Treatment Complexity

Polypharmacy also increases regimen complexity, which may compromise medication adherence. Older adults are often required to follow multiple dosing schedules, use different drug formulations, manage special administration instructions and coordinate prescriptions from several specialists. This burden is particularly problematic in patients with cognitive or visual impairment, depression, low health literacy, hearing loss, arthritis, reduced manual dexterity or limited social support [38,39].

Non-adherence may be intentional or unintentional. Intentional non-adherence may result from perceived adverse effects, fear of dependence, doubts about benefit or financial barriers. Unintentional non-adherence is often related to forgetfulness, complex regimens, poor understanding of instructions or difficulty in obtaining medications [40,41,42].

Medication safety should therefore not be evaluated solely through prescribing indicators. Treatment burden, adherence, treatment satisfaction, patient understanding and the feasibility of the regimen in everyday life are also clinically relevant outcomes. Medication review should assess whether the therapeutic regimen can realistically be followed by the patient or caregiver and whether its burden remains proportionate to its expected benefit.

### 3.3. Frailty, Cognitive Impairment and Functional Decline

Frailty is an important modifier of medication-related risk. It reflects reduced physiological reserve and increased vulnerability to stressors, but it is not synonymous with chronological age. Different operational approaches are used to identify frailty. The frailty phenotype characterises frailty through features such as unintentional weight loss, exhaustion, weakness, slow walking speed and low physical activity, whereas deficit-accumulation approaches quantify multiple health deficits across clinical and functional domains [43,44]. The relationship between frailty and polypharmacy is bidirectional. Frail individuals are more likely to have multimorbidity and therefore receive multiple medications, while polypharmacy may contribute to worsening frailty through adverse drug reactions, reduced mobility, anorexia, orthostatic hypotension, falls, fatigue and hospital-related functional decline [45].

Cognitive impairment further increases medication-related vulnerability. Patients with mild cognitive impairment or dementia may experience difficulties in understanding, remembering, and managing complex medication regimens safely [46]. At the same time, medications with anticholinergic or sedative properties may worsen cognition, increase delirium risk and impair balance [47,48,49,50]. Anticholinergic and sedative burden represent potentially modifiable contributors to cognitive and functional decline [49]; tools such as the Anticholinergic Cognitive Burden Scale and Drug Burden Index may help quantify cumulative exposure to drugs with anticholinergic and sedative effects, respectively, although differences between scales and the absence of universally accepted thresholds limit direct comparability [51,52].

Functional decline is particularly relevant in geriatric pharmacotherapy because preservation of independence may become a more important therapeutic objective than strict achievement of disease-specific targets. Medication-related dizziness, weakness, sedation, extrapyramidal symptoms, hypotension or hypoglycaemia may impair gait speed, balance, basic and instrumental activities of daily living [53,54,55]. In this context, medication review should not be limited to disease-specific targets but should also evaluate whether treatment supports or undermines independence, mobility and quality of life.

### 3.4. Falls, Hospitalisation and Transitions of Care

Polypharmacy is associated with increased risk of falls, fractures, emergency department visits, hospitalisation, institutionalisation and mortality [56].

Falls are among the most important geriatric consequences of polypharmacy. Fall-risk-increasing drugs include benzodiazepines, Z-drugs, antidepressants, antipsychotics, opioids, anticholinergics, antihypertensives, diuretics and glucose-lowering agents that can cause hypoglycaemia [57,58]. Exposure to several such medicines may further increase risk. However, much of the evidence linking medication exposure to falls is observational and may be influenced by multimorbidity, frailty and confounding by indication [59]. Medication review should therefore be considered as one component of multifactorial falls prevention, particularly after any fall, syncope, vertigo or delirium episode, or new decline in mobility [60,61].

Hospital admission itself may further increase medication burden through treatment intensification, therapeutic duplication, prescribing omissions, or incomplete medication reconciliation during transitions of care [62]. Transitions of care, particularly hospital discharge, are particularly high-risk, as older patients may receive new prescriptions while previous medications are unintentionally continued, discontinued or inadequately monitored.

Polypharmacy may also adversely affect the quality of life. Treatment burden, adverse effects, fear of falling, fatigue, gastrointestinal symptoms, sleep disturbance and reduced autonomy may significantly impair patient-reported outcomes. For many older adults, especially those with frailty or limited life expectancy, the main therapeutic priority may shift from disease prevention to preservation of function, comfort, cognition and independence [63,64].

### 3.5. Role of Geriatric Assessment in Medication Review

A comprehensive geriatric assessment (CGA) provides a relevant framework for medication review in older adults with polypharmacy. Unlike a classical medication review based solely on diagnoses or disease-specific guidelines, CGA integrates medical, functional, cognitive, psychological, nutritional and social domains. This multidimensional approach allows clinicians to evaluate the ongoing appropriateness of each medication in relation to the patient’s frailty status, functional reserve, life expectancy, treatment goals and personal preferences [33].

A CGA-based medication review should include medication reconciliation; assessment of indications, dosage and duration; identification of potentially inappropriate medications and prescribing omissions; evaluation of drug–drug and drug–disease interactions; and assessment of adherence and treatment burden. Treatment optimisation may include deprescribing, dose adjustment, therapeutic substitution, regimen simplification or additional monitoring, depending on the clinical context [34,65,66].

The medication review process is most effective when delivered by an interdisciplinary team, including geriatricians, clinical pharmacists, nurses, primary care physicians, specialists, caregivers and, whenever possible, the patient or their family. Such collaboration is particularly important during transitions of care, after hospitalisation and in patients with frailty, dementia, recurrent falls or advanced multimorbidity [14,15,16,67].

Overall, medication optimisation in geriatric practice should move beyond disease-specific prescribing towards an individualised, function-oriented and goal-concordant model of care. The objective is not simply to reduce medication count, but to ensure that treatment remains appropriate, clinically meaningful and aligned with what matters most to the older person [68,69]. This perspective may also help explain why improvements in prescribing indicators do not always translate directly into reductions in falls, hospitalisation, mortality or quality-of-life impairment, a question addressed in the following sections.

## 4. Structured Medication Review and Multidisciplinary Care

### 4.1. Structured Medication Review

The clinical foundation of polypharmacy management relies on structured medication review. The Pharmaceutical Care Network Europe (PCNE) defines medication review as a “*structured evaluation of a patient’s medicines with the aim of optimising medicines use and improving health outcomes*” [17]. In older adults, this process should include reconciliation of prescribed, over-the-counter, and complementary medicines; verification of indications; assessment of dose, duration, route, renal and hepatic adjustment; identification of therapeutic duplication; detection of drug–drug and drug–disease interactions; identification of potentially inappropriate medicines or unsafe complementary practices; detection of potential prescribing omissions; assessment of adherence; and formulation of a monitoring or deprescribing plan [17,18].

In 2018, the PCNE classified medication reviews into three main levels according to the information available to the reviewer. Type 1 is a basic prescription review based solely on the patient’s medication history. Type 2 reviews incorporate additional information from either the patient (Type 2A) or clinical records and laboratory data (Type 2B), allowing a more comprehensive assessment of medication-related problems. Type 3 represents the most comprehensive level of medication review, combining the patient’s medication history, a structured and in-depth patient interview, and relevant clinical and laboratory data [17]. This classification highlights an important practical issue: the quality and scope of medication review depend strongly on access to complete and clinically meaningful patient information.

The distinction between risk detection and risk resolution is essential. A screening tool may identify a drug as “*potentially inappropriate*”, but a clinician must determine whether its continued use is actually justified in relation to diagnosis, prognosis, frailty, renal and hepatic function, symptom burden, and patient goals and preferences [16,19,20]. For instance, anticoagulation may be appropriate in atrial fibrillation despite bleeding risk, whereas the same therapy may require reassessment in the presence of recurrent falls, advanced frailty, poor adherence, or severe renal impairment [70,71]. Similarly, antihypertensive treatment may be beneficial in robust older adults but may require adjustment when it contributes to orthostatic hypotension, syncope, or falls [30,72]. Medication review therefore requires clinical interpretation rather than mechanical application of prescribing rules. This assessment should explicitly incorporate cognition, functional status and life expectancy, ensuring that medication decisions remain aligned with goals of care and patient priorities rather than pharmacological risk alone.

Evidence from systematic reviews indicates that medication review interventions can improve prescribing appropriateness and reduce potentially inappropriate prescribing and medication-related problems, particularly when they use structured medication review, explicit prescribing criteria, pharmacist involvement, and/or multidisciplinary assessment [3,73]. However, effects on patient-level outcomes such as mortality, hospitalisation, falls, and quality of life appear less consistent [3,16,73]. Several factors may contribute to this discrepancy. Medication-review interventions differ substantially in setting, target population, professional involvement, access to clinical information, intensity, implementation of recommendations, and follow-up duration. In addition, many studies are powered primarily to detect changes in prescribing indicators rather than less frequent downstream clinical outcomes. Improvements in prescribing quality should therefore not be assumed to translate automatically into measurable clinical benefit.

Medication review should therefore be conceptualised not as a one-time intervention, but as a part of a longitudinal medication-safety pathway involving patient prioritisation, multidisciplinary decision-making, implementation of recommendations, documentation, follow-up, and reassessment over time [3,73]. These implementation dimensions are particularly relevant to pharmacist-led and multidisciplinary models, discussed in the following section.

### 4.2. Pharmacist-Led and Multidisciplinary Interventions

Pharmacists have a central role in polypharmacy management because medication-related problems frequently arise across multiple prescribers, dispensing records, and care settings. Pharmacist-led interventions and medication reviews can reduce potentially inappropriate prescribing and medication-related problems, particularly when they are based on structured tools, medication reconciliation, and multidisciplinary communication [31,32]. However, their effects depend strongly on the care setting, access to clinical information, professional responsibilities and the extent to which recommendations can be implemented.

Community pharmacists are well positioned to identify dispensing patterns, non-adherence, therapeutic duplication, over-the-counter medicines, dietary supplements, complementary therapies and high-risk combinations, especially in older adults receiving medicines from multiple prescribers [31,74]. Their contribution may nevertheless be limited when diagnostic information, laboratory values, frailty status or treatment goals are unavailable. Hospital pharmacists have a particularly important role in medication reconciliation at admission and discharge, when medication discrepancies are common and may contribute to preventable medication-related harm during transitions of care [15,32]. Clinical pharmacists embedded in geriatric or multidisciplinary teams can support deprescribing, dose adjustment, monitoring, patient counselling, and communication of medication changes across care settings [3,15,16].

The effectiveness of pharmacist-led medication review therefore depends on integration within comprehensive and coordinated care. Pharmacist expertise must be combined with diagnostic information, laboratory data, geriatric assessment, nursing observations, patient-reported outcomes, and patient priorities. Without access to this broader clinical context, pharmacists may identify possible medication-related risks but may be unable to determine whether a medicine is appropriate for a particular patient [15,16,17]. Conversely, prescribers may have limited visibility of dispensing behaviour, over-the-counter medicines, dietary supplements, complementary therapies, medication-taking difficulties, or cumulative exposure across therapeutic classes. Shared information infrastructure and interprofessional communication are therefore essential for meaningful medication optimisation [31,32,75].

Implementation remains a major challenge. The uptake of pharmacists’ recommendations depends on their clinical relevance and prioritisation, prescriber confidence, clarity of communication, patient preference, time availability, reimbursement mechanisms, and formal recognition of pharmacists’ clinical roles [31,75,76]. In healthcare systems where pharmacist-led medication review is not routinely reimbursed or integrated into established care pathways, implementation often remains dependent on local initiatives rather than becoming standard practice [3,75].

Importantly, pharmacist-led, multidisciplinary, transitional-care and primary-care medication reviews should not be considered equivalent interventions. They differ in timing, available information, professional authority, patient population and implementation context, which may partly explain variation in reported outcomes across studies. This heterogeneity should be taken into account when interpreting the evidence and when designing medication-review services for older adults. The main characteristics, evidence base, benefits, limitations, and implementation requirements of these intervention models are summarised in Table 1.

## 5. Explicit Prescribing Criteria and Risk-Assessment Tools

Explicit prescribing criteria provide reproducible methods for identifying potentially inappropriate medicines and potential prescribing omissions, but their scope, required clinical information and applicability differ across tools (Table 2). The STOPP/START criteria appear particularly relevant for pharmacotherapy assessment in European geriatric patients. STOPP identifies potentially inappropriate prescriptions, whereas START identifies omissions of clinically indicated therapy [19]. Version 3 includes 133 STOPP and 57 START criteria, developed through an international European Delphi process [19]. The criteria cover major therapeutic domains, including cardiovascular, antithrombotic, central nervous system, analgesic or endocrine medicines, also considering renal impairment, anticholinergic burden, duplicate prescribing, and fall-risk-increasing medicines. A particular strength of STOPP/START is that it addresses both overprescribing and undertreatment; however, many criteria require access to diagnoses, laboratory values and clinical context.

The AGS Beers criteria provide a complementary framework for identifying medications that should generally be avoided or used with caution in older adults [20]. They are widely used internationally and have extensive supporting evidence but were developed primarily for the United States. Differences in formularies, drug availability, prescribing practices, reimbursement, and regulatory context may therefore limit direct transferability to European healthcare systems. In European practice, Beers criteria are better interpreted as complementary to, rather than as a substitute for, locally or regionally adapted tools such as STOPP/START.

Belgium has contributed to practical community pharmacy screening through the Ghent Older People’s Prescriptions Community Pharmacy Screening tool (GheOP^3^S), originally developed using a RAND/UCLA appropriateness process (a structured consensus approach that combines available evidence with expert judgement to rate the appropriateness of clinical decisions when direct evidence is incomplete) [84] and was subsequently updated as GheOP^3^S version 2 to improve the detection of drug-related problems in older people in primary care [21]. This evolution illustrates the need for prescribing-screening tools to be periodically updated as evidence, prescribing patterns and clinical priorities change. Although the practical value of GheOP^3^S lies in real-world pharmacy screening, medication lists alone are insufficient to determine their appropriateness; diagnostic information, laboratory values, frailty status, treatment goals and patient preferences remain essential.

Other tools address more specific dimensions of medication optimisation. The EU (7)-PIM list, developed by a Delphi process involving seven European nations, provides a European list of potentially inappropriate medicines for older people and is particularly useful for prescribing audits and pharmacoepidemiological research [85]. Anticholinergic burden scales quantify cumulative exposure to medicines with antimuscarinic activity [51,52]. The Medication Appropriateness Index (MAI), by contrast, is an implicit, structured judgement-based tool that assesses indication, effectiveness, dosage, directions, interactions, duplication, duration, and cost [86]. More recently, the Potentially Inappropriate Prescribing Cascades (PIPC) list was developed through an international Delphi consensus process to identify prescribing cascades that may be inappropriate in older adults, thereby complementing medication review by focusing on adverse drug reactions misinterpreted as new conditions and followed by additional prescribing [87]. The Fit fOR The Aged classification (FORTA), originally developed in Germany, is a disease-specific positive–negative list that categorises medicines for older adults according to their suitability, ranging from class A, indispensable medicines, to class D, medicines that should generally be avoided [88]. Unlike purely negative lists of potentially inappropriate medicines, FORTA also supports the selection of appropriate therapeutic alternatives. Its clinical relevance is supported by the VALFORTA randomised trial, which showed that FORTA-guided prescribing improved medication appropriateness in hospitalised older adults with multimorbidity and polypharmacy [89]. Renal dose-adjustment guidance and interaction-screening systems address additional clinically important risks related to drug accumulation, toxicity and unsafe drug combinations [12,90].

These tools should therefore not be regarded as interchangeable. STOPP/START is particularly useful for comprehensive geriatric prescribing review; Beers criteria provide broad screening of high-risk medicines; GheOP^3^S is adapted to community-pharmacy practice; EU(7)-PIM is useful for European prescribing surveillance; FORTA supports disease-specific therapeutic selection; and the MAI enables individualised assessment of medication appropriateness. Choice of tool should depend on the clinical question, healthcare setting, information available and intended use. Importantly, identification of a potentially inappropriate medicine represents a signal for clinical review rather than an automatic indication for discontinuation. Prognosis, frailty, renal function, cognition, therapeutic goals, competing indications and patient preferences remain essential to final decision-making.

## 6. Deprescribing: A Tool for Medication Optimisation

Deprescribing is the planned and supervised reduction or discontinuation of medicines when potential harms outweigh benefits, when therapeutic objectives have changed, or when treatment is no longer aligned to prognosis, frailty status, or patient preferences [22]. It should not be understood as simple medication reduction, but as one component of medication optimisation. The aim is to improve appropriateness, reduce treatment burden, and prevent medication-related harm while preserving therapies that remain beneficial.

A structured deprescribing process generally involves identifying candidate medicines, assessing benefit–harm balance, prioritising drugs for withdrawal, shared decision-making, agreeing on a plan with the patient or caregiver, tapering when required, monitoring for withdrawal effects or recurrence of symptoms, and documenting the decision across care settings [22]. Drug classes frequently considered for deprescribing include benzodiazepines, Z-drugs, anticholinergics, antipsychotics, long-term proton-pump inhibitors without clear indication, duplicate antihypertensives, non-steroidal anti-inflammatory drugs, opioids, and hypoglycaemic agents in patients at high risk of hypoglycaemia. Deprescribing may be particularly challenging when the original indication is unclear, when treatment was initiated by another prescriber, or when withdrawal may lead to symptom recurrence or physiological rebound [91,92]. Shared decision-making and an appropriate monitoring plan are therefore essential.

Measuring deprescribing success remains complex. Medication count reduction alone is not an adequate endpoint because it may penalise appropriate polypharmacy, ignore correction of undertreatment and fail to capture whether medication changes improve outcomes that matter to older adults [3,22]. More relevant outcomes include reduction in potentially inappropriate medicines, lower anticholinergic or sedative burden, fewer drug-related problems, improved adherence, reduced treatment burden, fewer falls or adverse drug events, improved functional status, and patient-reported goal attainment [3,73]. The field therefore needs outcome frameworks that distinguish “*medication minimisation*” from “*medication optimisation*”. Medication burden, treatment satisfaction, adherence, quality of life, functional independence and attainment of patient-defined treatment goals should therefore be evaluated alongside prescribing safety.

The evidence for deprescribing remains mixed and depends on the outcome considered. Systematic reviews indicate that deprescribing and medication-review interventions can reduce potentially inappropriate prescribing and medication-related problems, but their effects on patient-level outcomes such as quality of life, falls, adverse drug events, hospitalisation, and mortality are less consistent [3,73]. This variability is also reflected in pragmatic trials. For example, a bundled pharmacist-led hyperpolypharmacy deprescribing intervention in older adults taking ten or more prescription medicines did not significantly reduce medication count or geriatric-syndrome outcomes compared with an integrated care setting in which deprescribing practices were already established [93]. This finding suggests that the additional effect of a deprescribing intervention may depend strongly on baseline care processes and the existing level of medication optimisation. More broadly, inconsistent findings may partly reflect heterogeneity in patient populations, care settings, baseline prescribing quality, targeted drug classes, patient engagement, professional roles, tapering support, implementation of recommendations, and follow-up duration [3,73,91]. Many studies are also primarily designed to detect changes in prescribing indicators rather than less frequent downstream clinical outcomes.

Implementation barriers may further limit effectiveness in routine practice. Patients and caregivers may be reluctant to discontinue long-standing medicines because of concerns about symptom recurrence or loss of perceived benefit, while clinicians may face uncertainty regarding withdrawal effects, responsibility for medicines initiated by another prescriber, or the appropriate monitoring strategy. Fragmented care, limited follow-up, time constraints and reimbursement mechanisms may also hinder deprescribing [22,91,92].

Deprescribing should therefore be evaluated as one component of longitudinal medication optimisation, rather than as a medication minimisation or a one-time reduction in the number of prescribed drugs.

## 7. European Implementation Perspectives: Examples from Belgium and Poland

European implementation initiatives have moved polypharmacy management from isolated medication reviews towards system-level medication-safety approaches. Notably, the EU-funded SIMPATHY initiative framed inappropriate polypharmacy and non-adherence as patient-safety challenges requiring policy alignment, professional education, risk stratification, patient involvement, and practical implementation tools [18]. iSIMPATHY subsequently extended this approach through pharmacist-led, person-centred medication reviews using a seven-step methodology across primary care, hospital, and outpatient settings [94].

Belgium and Poland are presented as illustrative rather than representative European healthcare systems, showing how medication-optimisation strategies depend on organisational and implementation context. Their comparison highlights two complementary implementation dimensions: Belgium provides experience with structured community-pharmacy medication review and reimbursement mechanisms, whereas Poland combines population-level pharmacoepidemiological capacity with an evolving framework for pharmacist involvement in individual medication management.

Belgium provides a relevant example of the progressive integration of community pharmacists into medication optimisation. The OptiMEDs intervention was designed to support multidisciplinary medication review in nursing homes through collaboration between general practitioners, community pharmacists and nurses, targeting pharmacotherapy optimisation among frail older residents [24]. Belgium has also contributed to the development of community-pharmacy screening through GheOP^3^S [21,84]. The SIMENON project demonstrated the feasibility and added value of structured, pharmacist-led medication review in community pharmacies and contributed to the development of a reimbursed medication review service [95,96]. Since 1 April 2023, Belgian community pharmacists have been reimbursed for medication reviews intended for patients taking at least five chronic reimbursed medicines [97,98]. In addition, resources from the Belgian Centre for Pharmacotherapeutic Information (CBIP) provide specific guidance on medication review and rational prescribing in older adults, including dedicated resources for patients aged 80 years and older [97,98].

Implementation nevertheless remains constrained by patient refusal, time and staffing constraints, inadequate consultation spaces, privacy-compliant communication requirements, limited awareness among general practitioners and patients, and insufficient interprofessional collaboration [99,100]. Access to relevant clinical information also remains a critical issue: medication appropriateness cannot always be assessed from dispensing data alone when diagnoses, laboratory values, frailty status, treatment goals or patient preferences are unavailable. These constraints make risk prioritisation and data integration central to the scalability of pharmacist-led medication review.

Poland offers a complementary perspective through large-scale pharmacoepidemiological capacity. National Health Fund data enable population-level analysis of prescribing patterns, age-related medication burden, and chronic polypharmacy [7,101], while population-based studies such as PolSenior provide additional geriatric and medication-use information [8]. Pharmaceutical care has also been formally recognised within the Polish legal framework as a healthcare service delivered by pharmacists in collaboration with patients, physicians and, when necessary, other healthcare professionals [102]. A national pilot programme for medication reviews was subsequently established, including older adults with substantial medication burden, illustrating efforts to translate pharmaceutical care into structured medication-review practice [103]. Polish resources also include adapted Beers and STOPP/START criteria for geriatric pharmacotherapy [104]. This creates opportunities for identifying high-risk groups, monitoring prescribing patterns, and strengthening medication safety. However, population-level detection of polypharmacy does not automatically translate into patient-level medication optimisation. The key implementation challenge lies in connecting epidemiological signals from reimbursement and dispensing databases with clinical workflows involving primary care physicians, pharmacists, nurses, patients, and caregivers.

The comparison is intended to highlight context-dependent implementation rather than to rank healthcare systems. Differences in primary-care organisation, reimbursement, professional roles, access to clinical data, pharmacist-prescriber collaboration, and digital infrastructure may substantially influence feasibility and effectiveness. Therefore, lessons from Belgium and Poland should not be assumed to transfer directly to other European settings. Table 3 summarises the main strengths, limitations and implementation opportunities in the two settings.

## 8. Digital Decision Support and AI-Enabled Pharmacovigilance

### 8.1. Why Post-Marketing Pharmacovigilance Is Particularly Important in Older Adults

The limited representativeness of older adults in pre-authorisation clinical trials is a major rationale for strengthened pharmacovigilance in geriatric medicine. Very old, frail and multimorbid patients, particularly those exposed to polypharmacy, may be less well represented than the populations who subsequently receive these medicines in routine clinical practice [105,106,107,108]. The generalisability of pre-marketing safety data is clearly limited, because age-related changes in pharmacokinetics and pharmacodynamics, renal and hepatic impairments, frailty, drug–drug and drug–disease interactions, and complex multidrug regimens may modify both drug exposure and susceptibility to adverse effects. Regulatory guidance therefore emphasises the inclusion of patients aged 75 years and older, as well as patients with concomitant diseases and medications, in clinical development programmes [107,108].

Moreover, the safety profile of a newly authorised medicine cannot be considered fully characterised at the time of approval as clinical trials are usually conducted in highly selected populations of patients. Rare, delayed or context-dependent adverse reactions may become apparent only after exposure of larger and more heterogeneous patient populations, particularly in routine care where multimorbidity and concomitant treatments are common [109,110]. Continuous post-marketing pharmacovigilance is therefore particularly important in older adults and should complement pre-authorisation evidence. Spontaneous adverse-reaction reporting systems, including EudraVigilance and FAERS, together with active surveillance based on electronic health records, claims data and longitudinal clinical information, provide complementary opportunities for detecting emerging medication-safety signals [109,110,111]. Such signals require clinical assessment and validation, because spontaneous reports alone cannot establish causality or provide direct estimates of adverse-event incidence [109].

### 8.2. From Rule-Based Clinical Decision Support to AI-Enabled Pharmacovigilance

Digital medication safety tools have traditionally relied on rule-based clinical decision support systems embedded in electronic prescribing, pharmacy dispensing, and electronic health record platforms. These systems use predefined rules, drug databases, and explicit prescribing criteria to identify contraindications, therapeutic duplication, renal dosing problems, known drug–drug interactions, and potentially inappropriate medicines [19,20,25]. Their strengths are transparency, interpretability, and direct linkage to established clinical rules. However, rule-based systems are often limited by alert fatigue, poor prioritisation, incomplete clinical context, and insufficient integration of frailty, prognosis, multimorbidity, treatment burden, patient preferences, and competing therapeutic goals [112,113].

AI-enabled methods may extend conventional decision support by integrating heterogeneous data sources and estimating patient-specific medication-related risk. Machine-learning models can combine prescriptions, diagnoses, laboratory values, renal and hepatic function, hospitalisation history, falls, frailty proxies, dispensing data, adherence indicators, anticholinergic burden, sedative burden, and care-transition events to predict adverse drug events, bleeding, delirium, hypoglycaemia, falls, or hospitalisation [25,26,27,28,29,114]. Natural language processing may further support pharmacovigilance by extracting suspected adverse drug events, discontinuation reasons, medication changes, symptoms, adherence barriers, falls, delirium episodes, and functional decline from unstructured electronic health record notes [27]. Knowledge graphs and graph neural networks can model relationships between drugs, targets, diseases, phenotypes, and adverse effects, supporting more sophisticated detection of potential drug–drug and drug–disease interactions [28,29].

### 8.3. AI for Personalised Medication-Risk Prediction and Clinical Prioritisation

In pharmacovigilance, AI may support signal detection, case triage, duplicate detection, literature screening, active surveillance, and prioritisation of reports requiring human review [25,27,115]. For geriatric polypharmacy, the most plausible near-term role of AI is not autonomous prescribing or autonomous deprescribing, but clinical prioritisation and decision support by identifying patients at highest risk, ranking medication-related contributors, detecting less frequent but clinically relevant adverse drug events, and supporting pharmacist-led medication review within multidisciplinary care. This prioritisation function is particularly relevant because systematic medication review of all older adults with polypharmacy would be difficult to sustain at scale. AI-supported risk stratification could therefore help direct limited clinical and pharmacy resources towards patients most likely to benefit from comprehensive medication review.

AI-supported deprescribing remains an emerging field. In principle, predictive models could help estimate which patients are most likely to benefit from medication reduction, which medicines contribute most to risk, and what monitoring plan is needed upon withdrawal. However, deprescribing decisions are preference-sensitive and clinically contextual. An algorithm may identify a benzodiazepine, anticholinergic medicine, or duplicate antihypertensive as high risk, but only a clinician–patient process can determine whether deprescribing is acceptable, how tapering should occur, and what symptoms or adverse effects should be monitored [22,23].

The geriatric risks described above provide clinically meaningful targets for AI development. Models should not only predict generic hospitalisation risk; they should identify modifiable and interpretable pathways, notably: (i) anticholinergic burden contributing to delirium; (ii) sedative burden contributing to falls; (iii) renal decline increasing exposure to high-risk medicines; (iv) serotonergic toxicity, including serotonin syndrome, arising from high-risk drug combinations; or (v) poor adherence contributing to treatment failure. Importantly, AI-enabled pharmacovigilance should be designed around clinically interpretable risk pathways and actionable contributors rather than opaque global risk scores [112,114,116].

### 8.4. Methodological, Regulatory, and Ethical Challenges

Several methodological barriers limit AI deployment in geriatric medication safety, and AI tools require rigorous evaluation before clinical deployment. Retrospective predictive performance is not equivalent to clinical utility. Models may be affected by missing or incomplete data, coding bias, under-reporting of adverse drug events, uncertain causal links, incomplete capture of over-the-counter medicines or dietary supplements, confounding by indication, imbalanced outcome classes, and poor generalisability across populations or healthcare systems [27,114,115]. In addition, the integration of AI tools into routine practice may be hindered by limited interoperability and data fragmentation across hospital information systems, primary-care records, community pharmacy software, nursing-home systems and electronic health platforms. Relevant information may therefore remain incomplete or inaccessible, including diagnoses, laboratory values, frailty indicators, treatment goals, patient preferences, over-the-counter medicines and dietary supplements. In the absence of sufficiently integrated and up-to-date data, AI-based risk stratification may be incomplete, poorly actionable or difficult to embed in routine medication-review workflows. In particular, missing medication histories, undocumented over-the-counter use, fragmented care records and coding errors may systematically distort model inputs; the completeness and representativeness of training and deployment data should therefore be assessed explicitly.

Furthermore, the lack of explainability of some AI models may reduce clinician trust and acceptance, particularly when recommendations influence high-stakes prescribing or deprescribing decisions. In older adults, additional challenges include heterogeneity in frailty and multimorbidity, cognitive impairment, institutionalisation and underrepresentation of clinically complex populations in training datasets. AI systems should therefore also be evaluated for differential performance across clinically relevant subgroups, particularly very frail older adults, people with cognitive impairment, nursing-home residents and socioeconomically or geographically diverse populations. Natural language processing models may perform differently across languages, documentation styles, and electronic health record systems. Model performance may further deteriorate over time as prescribing practices, formularies, clinical guidelines, coding systems and patient populations evolve. Prospective evaluation should therefore assess not only discrimination, but also calibration, external validity, subgroup performance and the need for post-deployment monitoring and recalibration. In medication safety, false-positive predictions or alerts may contribute to alert fatigue and unnecessary review, whereas false-negative predictions may fail to identify clinically important medication-related harm. Drug–drug interaction models may predict plausible interactions without estimating clinical severity, preventability, or actionability. Changes in disease prevalence should also be considered as a source of temporal dataset shift. Ongoing performance surveillance should trigger recalibration or external revalidation when clinically meaningful drift is detected.

Regulatory and ethical considerations are central to implementation. AI systems used for medication-risk management may fall within medical-device or clinical decision-support regulation when they generate patient-specific recommendations. They must comply with data-protection principles, including purpose limitation, data minimisation, security, auditability, and transparency. Human oversight is essential, especially when recommendations concern high-risk medicines such as anticoagulants, opioids, psychotropics, hypoglycaemic agents, or cardiovascular drugs. AI systems for medication safety should therefore be explainable, externally validated, calibrated and auditable, and should be integrated into accountable clinical workflows. Their role should be to support clinical interpretation and professional judgement rather than replace pharmacist, geriatric or multidisciplinary decision-making.

## 9. Critical Analysis, Knowledge Gaps, and Limitations

Despite substantial progress, several gaps limit the translation of polypharmacy research into routine geriatric care. First, the field remains heterogeneous in definitions and outcomes. Studies variously assess medication count, potentially inappropriate medicines, drug-related problems, quality of life, adherence, treatment burden, adverse drug reactions, frailty, falls, hospitalisation, and/or mortality [1,2,3,4]. Definitions of medication exposure also vary between studies, further complicating comparison. This heterogeneity makes it difficult to compare interventions and to determine which outcomes best reflect meaningful patient benefit. Medication count is useful for identifying risk, but it does not distinguish appropriate from inappropriate polypharmacy.

Second, evidence is stronger for improving prescribing indicators than for improving clinical outcomes. Medication reviews frequently reduce potentially inappropriate prescribing and medication-related problems, but their effects on quality of life, falls, hospitalisation, and mortality are less consistent [3,16]. This does not necessarily imply a lack of clinical value; indeed, medication optimisation may prevent rare but severe outcomes that require large samples and long follow-up to demonstrate. It may also generate benefits such as lower treatment burden, improved patient understanding, better adherence, or greater therapeutic coherence, which are not always captured by conventional clinical endpoints.

Third, explicit criteria and screening tools are valuable but incomplete. STOPP/START, Beers, GheOP^3^S, EU (7)-PIM, FORTA, anticholinergic burden scales, renal function-based dose-adjustment guidance and the Medication Appropriateness Index improve detection of medication-related problems, but they cannot fully incorporate prognosis, frailty, cognitive status, functional reserve, therapeutic goals, competing indications, or patient preferences [12,19,20,51,84,85,86,88]. These tools should therefore be used as structured prompts for clinical judgement rather than as automatic decision rules.

Fourth, deprescribing remains difficult to implement and evaluate. Medication count reduction alone is insufficient because it may penalise appropriate polypharmacy, overlook undertreatment, and miss outcomes that matter to older adults, such as reduced treatment burden, fewer drug-related problems, and better functioning with improved quality of life [3,22,73,93]. Barriers include patient and caregiver concerns, clinician uncertainty, fragmented responsibility, limited follow-up and reimbursement constraints [75,91,92]. Deprescribing is especially challenging when the original indication is unclear, when medicines were initiated by another prescriber, or when symptom recurrence or withdrawal effects are expected after discontinuation. It therefore requires shared decision-making, a tapering and monitoring plan when appropriate, and clear communication across professionals, rather than only identification of candidate medicines [22,91,92].

Fifth, adverse drug reactions and prescribing cascades remain under-recognised in frail older adults. Symptoms such as dizziness, confusion, fatigue, anorexia, urinary retention, falls, delirium or functional decline may be misattributed to ageing or disease progression, leading to therapeutic escalation rather than medication optimisation [13,35,117]. Comprehensive geriatric assessment should therefore be integrated into medication review, especially in patients with frailty, cognitive impairment, recurrent falls, renal impairment or recent hospitalisation [33,34]. This problem is compounded by the limited representation of very old, frail and multimorbid patients in pre-authorisation trials, creating residual uncertainty about medication safety in the populations at greatest risk of treatment-related harm. Post-marketing and age-stratified pharmacovigilance should therefore be considered an essential complement to conventional efficacy and safety evidence.

Sixth, Belgium and Poland illustrate complementary implementation challenges. Belgium has medication-review tools and reimbursed pharmacist-led medication reviews, but implementation remains limited by fragmented data and incomplete access to clinical context [6,24,84,118]. Poland has strong National Health Fund data for population-level monitoring, but reimbursement data do not capture the full medication burden, particularly over-the-counter medicines, non-reimbursed medicines and dietary supplements [7,101]. In both countries, the key challenge is translating available medication-risk information into clinically actionable, patient-level medication optimisation.

Finally, AI offers promising opportunities for risk stratification, adverse-event detection, drug–drug interaction prediction, pharmacist-led review prioritisation and pharmacovigilance [25,27,28,29,112,113,114,115]. However, predictive accuracy is not equivalent to clinical utility. AI models may be limited by missing data, coding bias, under-reporting, confounding, poor generalisability, underrepresentation of frail or cognitively impaired older adults, and insufficient access to linked, up-to-date and clinically meaningful data. Fragmentation across hospitals, general practitioners, pharmacies, nursing homes and eHealth platforms may further reduce the completeness and actionability of AI-supported risk assessment. Training datasets should adequately represent the diversity of older populations, since underrepresentation of women, ethnic and cultural minority groups, socioeconomically disadvantaged individuals, or populations from different geographical regions may introduce algorithmic bias and lead to unequal model performance across patient groups. External validation, subgroup evaluation and prospective assessment are therefore essential before widespread clinical implementation.

As a narrative review, the present synthesis also has methodological limitations. It was designed to integrate clinically important and conceptually diverse evidence rather than to provide exhaustive study identification, formal risk-of-bias assessment across all included sources, or pooled effect estimates. The breadth of topics—spanning geriatric pharmacotherapy, health-system implementation, and rapidly evolving AI methods—also means that the maturity and comparability of the underlying evidence vary considerably. Conclusions regarding emerging AI applications should therefore be interpreted as an assessment of current opportunities and limitations rather than evidence of established predictive accuracy or clinical effectiveness.

## 10. Proposed Conceptual Framework for Personalised Medication Safety

**Figure 2:**
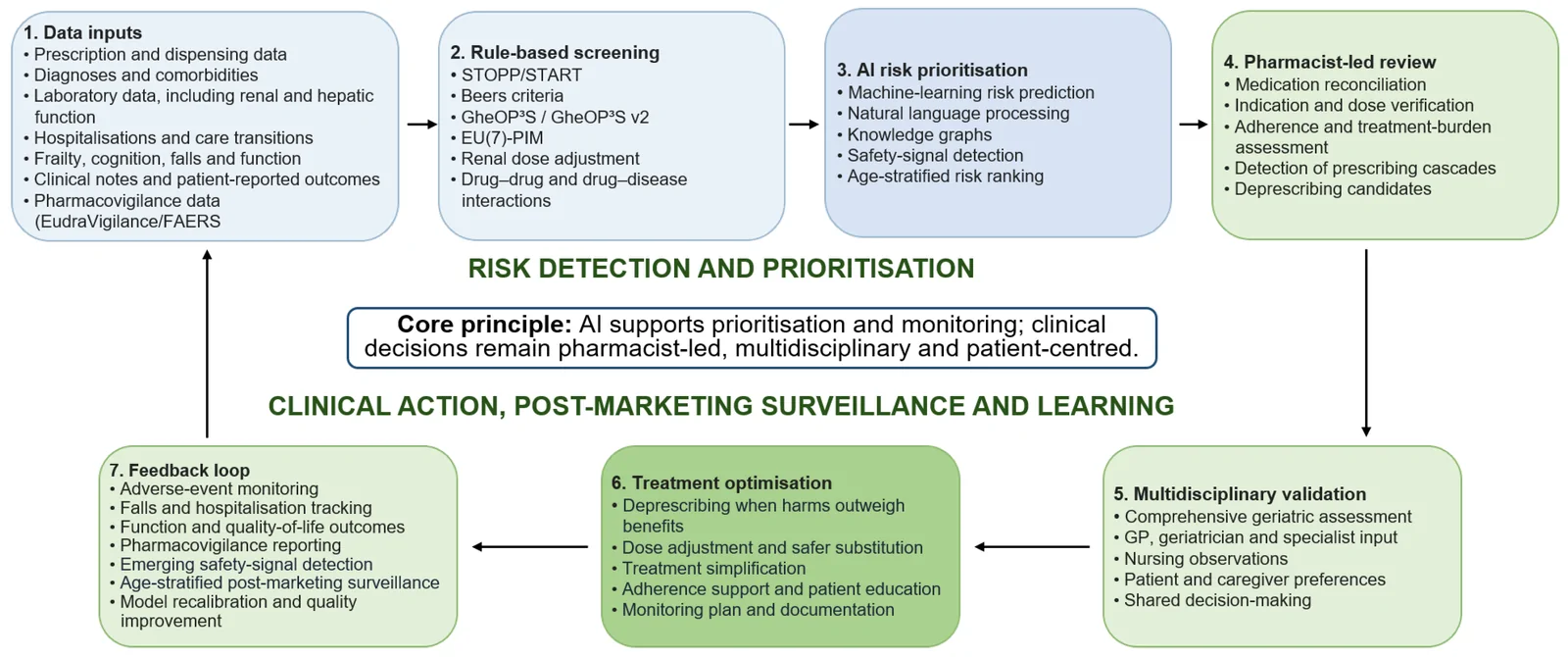
Proposed conceptual framework for closed-loop AI-enabled pharmacovigilance and personalised medication-risk management in older adults with polypharmacy. The model integrates heterogeneous data sources, explicit prescribing criteria, AI-based risk stratification, pharmacist-led medication review, comprehensive geriatric assessment, multidisciplinary decision-making, treatment optimisation, and longitudinal pharmacovigilance feedback. Blue boxes represent data inputs, rule-based screening and AI-supported risk detection and prioritisation; green boxes represent pharmacist-led and multidisciplinary clinical review, treatment optimisation, and feedback/learning. AI, artificial intelligence; STOPP, Screening Tool of Older Persons’ Prescriptions; START, Screening Tool to Alert to Right Treatment; GheOP^3^S, Ghent Older People’s Prescriptions Community Pharmacy Screening; EU(7)-PIM, European Union (7)-Potentially Inappropriate Medications; GP, general practitioner; FAERS, FDA Adverse Event Reporting System. Arrows indicate the proposed direction of the medication-review workflow.

On the basis of the clinical, organisational and technological gaps identified in this review, a closed-loop medication-safety framework can be proposed to connect risk detection with clinical interpretation, treatment optimisation and longitudinal monitoring (Figure 2). The framework is based on the principle that medication safety in older adults cannot be achieved by a single tool or professional discipline. Explicit prescribing criteria improve consistency of risk detection, AI may support prioritisation in large and heterogeneous populations, pharmacists contribute detailed medication expertise, and comprehensive geriatric and multidisciplinary assessment provides the clinical context needed to determine whether an identified risk is actionable. Patient and caregiver preferences are then required to translate this assessment into treatment decisions that are clinically appropriate and goal-concordant.

The first layer consists of data integration. Relevant information may arise from prescriptions, dispensing records, diagnoses, laboratory results, hospitalisations, care transitions, frailty indicators, cognitive and functional status, falls history and clinical notes. This layer is essential because neither rule-based screening nor AI-based prediction can generate clinically meaningful outputs when important medication or patient information is missing, outdated or fragmented across care settings.

The second layer applies structured rule-based screening, including explicit prescribing criteria, renal dose-adjustment guidance, drug–drug and drug–disease interaction screening, and assessment of anticholinergic and sedative burden. These tools provide transparent and clinically interpretable signals, but they are intentionally positioned as a screening layer rather than as a decision layer because they cannot fully account for prognosis, frailty, competing therapeutic priorities or patient preferences.

The third layer introduces AI-supported risk prioritisation. Its purpose is not to replace explicit criteria, but to complement them by integrating multiple risk factors and ranking patients or medication-related problems according to their estimated clinical relevance. This prioritisation step is particularly important in healthcare systems where comprehensive medication review cannot feasibly be performed for every older adult with polypharmacy. AI may therefore help direct clinical resources towards patients most likely to benefit from more detailed review.

The fourth layer consists of pharmacist-led medication review. At this stage, medication-related signals are interpreted in relation to indication, dosage, duration, adherence, duplication, interactions, monitoring requirements and treatment burden. Pharmacists can determine whether alerts generated by screening or AI are pharmacologically plausible, clinically relevant and potentially modifiable, thereby reducing the risk of mechanistic or low-value recommendations.

The fifth layer provides multidisciplinary clinical validation, integrating comprehensive geriatric assessment with diagnostic information, frailty, cognition, functional status, prognosis, treatment goals and patient or caregiver preferences. This layer is necessary because medication appropriateness in older adults cannot be determined from pharmacological risk alone. A medicine identified as potentially inappropriate may still be clinically justified, while a medicine not flagged by an explicit criterion may nevertheless be inappropriate in the context of advanced frailty, limited life expectancy or changing treatment priorities.

The sixth layer translates validated findings into treatment optimisation, including deprescribing, dose adjustment, therapeutic substitution, additional monitoring, adherence support or patient education. The objective is therefore not medication reduction per se, but alignment of treatment with clinical need, individual vulnerability and patient-defined goals [17,21,22,24,26,112].

Finally, the framework incorporates a feedback loop in which treatment changes and subsequent outcomes are fed back into pharmacovigilance and quality-improvement systems. Adverse drug events, successful deprescribing, symptom recurrence, hospitalisation, falls, treatment burden and patient-reported outcomes can therefore inform subsequent reassessment. This longitudinal component is important because medication optimisation is not a one-time intervention: medication-related risk evolves with disease progression, care transitions, new prescriptions and changes in functional or cognitive status.

The proposed framework therefore links three functions that are often considered separately: risk detection, clinical interpretation and treatment optimisation. Its central premise is that AI has greatest potential when embedded between structured risk detection and human clinical review, rather than when used as an autonomous prescribing or deprescribing system.

This framework represents an original conceptual synthesis proposed by the authors. It has not been empirically validated and should therefore be prospectively evaluated before being interpreted as an established model of care.

## 11. Future Directions and Research Priorities

Future research should move from isolated medication-review studies towards integrated, pragmatic and scalable medication-safety models. Randomised and quasi-experimental studies should compare usual care, structured pharmacist-led medication review, comprehensive geriatric assessment-integrated medication review, AI-assisted prioritisation and hybrid multidisciplinary approaches. Outcomes should extend beyond medication count and prescribing appropriateness to include prescribing omissions, adverse drug reactions, falls, hospitalisations, emergency consultations, adherence, treatment burden, quality of life, patient-reported outcomes and cost-effectiveness [3,22,73]. Frail older adults, nursing-home residents, cognitively impaired patients and socioeconomically vulnerable groups should be explicitly included to improve the applicability of findings to those most exposed to medication-related harm. Patient-centred endpoints should specifically include treatment satisfaction, functional independence and attainment of individual treatment goals.

AI development should focus on clinical usefulness rather than predictive performance alone. Models should identify not only whether a patient is at high risk, but also which medicines, combinations, physiological factors, geriatric syndromes or care-transition events contribute to that risk. Hybrid systems combining explicit prescribing criteria with machine-learning risk estimation may offer a clinically interpretable strategy. For example, STOPP/START or Beers criteria may generate transparent rule-based risk signals, while machine-learning models estimate patient-specific probability of adverse outcomes using laboratory values, prior events, frailty proxies, dispensing patterns and care-transition history [19,20,25,27,28,29,112,114]. Future studies should prospectively assess whether these approaches improve medication-related and patient-centred outcomes compared with existing care pathways. Evaluation should include discrimination, calibration, external validity and subgroup performance, together with post-deployment monitoring and recalibration when prescribing practices, formularies, coding systems, clinical guidelines or patient populations change over time. Broader AI and health-technology research also illustrates the potential of multiplex-network methods for analysing heterogeneous biomedical relationships and the importance of organisational networks for translating AI-enabled technologies into practice [119,120]. These studies are not specific to medication optimisation and should therefore be interpreted as contextual methodological and implementation evidence rather than direct evidence of clinical effectiveness.

At the European level, medication optimisation should align with the European Health Data Space, pharmacovigilance strategy, AI regulation and patient-safety priorities. The European Health Data Space creates a regulatory framework for improving access to electronic health data and supporting research, innovation, policymaking, patient safety and personalised medicine, while the EU AI Act establishes rules for safe and trustworthy AI systems, including high-risk applications affecting health and safety [121,122]. Future medication-safety tools should therefore be evaluated not only for predictive performance, but also for clinical safety, fairness, interpretability, cybersecurity, data protection, human oversight and real-world implementation. Cross-country studies using harmonised outcome definitions and comparable evaluation frameworks would be particularly valuable for determining whether AI-assisted medication review can be transferred safely across different healthcare systems.

At the national level, Belgium and Poland provide complementary opportunities for such research. Belgium has developed strong experience in multidisciplinary medication review, community-pharmacy tools and nursing-home interventions, but further progress depends on reducing data fragmentation across hospitals, general practitioners, pharmacies and nursing homes. Better integration of clinical, dispensing and laboratory data would facilitate the safe use of AI and machine-learning tools to support medication-risk stratification and shared decision-making. Poland, by contrast, has strong pharmacoepidemiological capacity through national prescribing and claims data, but this population-level intelligence could be more directly connected to clinical workflows. A useful Belgian–Polish research agenda would therefore test whether large-scale data can identify high-risk older adults and trigger pharmacist-led or multidisciplinary medication review, with evaluation of clinical outcomes, cost-effectiveness and scalability across European health-system contexts [7,21,24,84].

## 12. Conclusions

Polypharmacy in older adults is a multidimensional medication-safety challenge rather than a simple count of medicines. The central clinical distinction is between appropriate and inappropriate polypharmacy, assessed in relation to indication, frailty, cognition, functional status, prognosis, treatment burden and patient preferences. Structured medication review, pharmacist-led interventions, explicit prescribing criteria, deprescribing, and comprehensive geriatric assessment remain the foundation of geriatric medication optimisation.

European initiatives such as SIMPATHY and iSIMPATHY, together with Belgian and Polish implementation experience, illustrate both the opportunities and the system-level challenges involved in translating medication-risk detection into clinically meaningful action. AI-enabled pharmacovigilance and personalised risk management may improve risk detection, prioritisation, and longitudinal monitoring, but they should be integrated as decision-support tools rather than autonomous decision-makers. Their clinical value will depend on data quality and interoperability, explainability, external validation, prospective evaluation, human oversight and integration into pharmacist-led multidisciplinary workflows. AI-enabled medication optimisation should therefore be regarded as a promising adjunctive decision-support approach rather than an established standard of care; prospective validation, real-world implementation studies, safety monitoring and demonstrated patient benefit are required before widespread adoption.

The future of geriatric medication safety therefore lies in combining structured clinical judgement, patient-centred deprescribing, comprehensive geriatric assessment, interoperable health data, and accountable AI within longitudinal care pathways that support safer, more personalised, and more clinically meaningful medication use in older adults.

## Figures and Tables

**Table 1:** Major types of medication-optimisation interventions for older adults.

Intervention Type	Target Population	Healthcare Setting	Responsible Professional(s)	Principal Outcomes Measured	Evidence Strength *	Major Benefits	Main Limitations	Key Implementation Requirements
Pharmacist-led medication review	Older adults with polypharmacy, multimorbidity or high medication-related risk	Community pharmacy, primary care, hospital, long-term care	Pharmacist, usually with physician communication or referral	PIMs, DRPs, medication number, recommendations accepted, adherence; less frequently QoL, hospitalisation, falls or mortality	Moderate for prescribing/process outcomes; inconsistent for patient-level outcomes [3,77]	Detailed pharmacological expertise; identification of interactions, duplication, dosing problems and adherence barriers; feasible across several settings	Effect depends on access to clinical information and physician acceptance; heterogeneity in review intensity and follow-up	Access to medication and clinical records; protected time; communication with prescribers; referral pathway; reimbursement; follow-up
Multidisciplinary medication review	Older adults with multimorbidity, complex polypharmacy or frailty	Primary care, geriatric clinics, hospitals, long-term care	Physician + pharmacist ± nurse, geriatrician or other professionals	Medication appropriateness, DRPs, medication number, adherence, function, cognition, falls, admissions, QoL	Moderate for medication appropriateness; low/inconsistent for clinical outcomes [78]	Allows pharmacological decisions to be integrated with diagnoses, frailty, functional status and care goals	Resource intensive; heterogeneous team composition and intervention intensity; benefits depend on effective collaboration	Defined roles; shared records; team communication; structured review process; patient/caregiver involvement; longitudinal follow-up
Computerised clinical decision support (CDSS)	Older adults receiving potentially inappropriate or complex medication regimens	EHR-enabled primary care and hospital settings	Prescriber/pharmacist supported by electronic system	PIM initiation, deprescribing, alert response, ADEs	Moderate-certainty evidence for reducing some PIM prescribing; lower certainty for ADE reduction [79]	Scalable; consistent screening; can incorporate explicit criteria, laboratory data and drug interactions	Alert fatigue, clinician non-adherence, incomplete EHR data, interoperability problems and variable usability	High-quality structured data; EHR integration; clinically validated rules; appropriate alert design; clinician oversight; performance monitoring
Transitional-care medication reconciliation and review	Older patients undergoing admission, ward transfer, hospital discharge or transfer to another care setting	Hospital-primary care, hospital-community pharmacy, hospital-long-term care interfaces	Pharmacist, physician, nurse or multidisciplinary team	Medication discrepancies, continuity of medication changes, ADEs, adherence, readmissions, ED use, patient-reported outcomes	Moderate for reducing discrepancies/improving continuity; inconsistent for hard clinical outcomes [80]	Addresses a period of particularly high medication risk; can identify omissions, unintended changes, duplication and communication failures	Often multi-component, making attribution difficult; responsibility may be fragmented between settings; benefits may decay after discharge	Best possible medication history; reconciliation at each transition; clear discharge medication list and rationale for changes; patient/caregiver counselling; rapid communication with community clinicians; post-discharge follow-up
Primary-care medication review/deprescribing	Community-dwelling older adults with polypharmacy or potentially inappropriate treatment	General practice/primary care	GP/family physician, frequently supported by pharmacist or nurse	Medication number, PIMs, DRPs, falls, hospitalisation, adherence, QoL and function	Moderate for medication reduction and appropriateness; emerging but inconsistent for clinical outcomes [81]	Longitudinal knowledge of the patient; opportunity to integrate multimorbidity, preferences and preventive treatment goals	Time constraints; fragmented specialist prescribing; unclear ownership of individual medicines; deprescribing may require repeated discussions	Scheduled review; access to specialist and hospital medication information; patient involvement; follow-up and monitoring after changes
Frailty- and goals-of-care-directed review	Patients with severe frailty, limited life expectancy or high treatment burden	Geriatric, long-term-care and palliative-care settings	Physician/geriatrician with pharmacist and palliative-care input as appropriate	Medication burden, symptom burden, QoL, treatment satisfaction, achievement of patient goals and discontinuation of low-benefit preventive treatment	Evidence supports deprescribing feasibility; evidence for hard endpoints remains limited [82,83]	Aligns treatment with time-to-benefit, prognosis and patient priorities; may reduce unnecessary treatment burden	Prognostic uncertainty; risk of inappropriate withdrawal if goals and indications are insufficiently assessed	Frailty, cognition, function and prognosis assessment; explicit goals-of-care discussion; shared decision-making; documentation and monitoring

* The terms describing evidence strength represent a qualitative synthesis of recent reviews and should not be interpreted as formal GRADE ratings except where the cited review specifically used GRADE methodology.

**Table 2:** Major rule-based medication-screening and risk-assessment tools used in medication review for older adults with polypharmacy.

Tool/Criterion	Main Purpose	Type of Risk Detected	Main Advantage	Key Limitation	Applicability/Setting
STOPP/START criteria [19]	Identification of potentially inappropriate prescriptions and prescribing omissions across major therapeutic domains	Inappropriate medicines, missing indicated therapies, duplicate prescribing, fall-risk drugs, renal-risk prescribing	Addresses both overprescribing and undertreatment; strong European relevance; incorporates several clinically important risk domains	Many criteria require diagnoses, laboratory data and clinical context; does not replace individual benefit–risk assessment	Particularly applicable to comprehensive geriatric medication review in hospital, outpatient and primary-care settings
AGS Beers Criteria [20]	Identification of medicines that should generally be avoided or used with caution in older adults, including drug–disease and renal-risk prescribing	High-risk medicines, drug–disease interactions, renal-risk medicines, medicines requiring caution	Widely recognised; broad coverage; relatively straightforward for screening and research	Developed primarily for the US; differences in formularies and prescribing practice may limit direct transferability	Widely used in research and clinical screening, but requires adaptation when applied outside North America
GheOP^3^S/GheOP^3^S v2 [21,84]	Detection of potentially inappropriate prescribing and drug-related problems in older adults	Drug-related problems, potentially inappropriate medicines, interactions, renal-risk prescribing	Designed for practical community-pharmacy use; relevant to Belgian and European primary care	Medication-list information alone may be insufficient; clinical and laboratory information may be required for interpretation	Particularly suited to community-pharmacy and primary-care medication review
EU(7)-PIM list [85]	Identification of potentially inappropriate medicines in older adults	Medicines with unfavourable benefit–risk profiles in older adults	European focus; useful for prescribing audits, epidemiological studies and cross-country comparisons	Primarily identifies medicines to avoid; less emphasis on prescribing omissions or individual clinical context	Particularly useful for European pharmacoepidemiological research and prescribing surveillance
MAI [86]	Patient-specific assessment of indication, effectiveness, dose, directions, interactions, duplication, duration and cost	Inappropriate indication, dose, directions, interactions, duplication or duration	Goes beyond fixed PIM lists; allows individualised clinical judgement	Time-consuming; requires detailed patient information and trained clinical judgement	Best suited to comprehensive clinical or research-based medication review rather than rapid population screening
PIPC list [87]	Identification of potentially inappropriate prescribing cascades in older adults	Drug therapy initiated to treat an adverse drug reaction caused by another medicine	Specifically targets prescribing cascades that may be missed by conventional PIM criteria	Requires clinical interpretation to establish whether the sequence represents an inappropriate cascade	Useful as a complementary tool in geriatric, pharmacy and multidisciplinary medication review
FORTA [88,89]	Disease-specific classification of medicines according to suitability in older adults	Both inappropriate and appropriate therapeutic choices	Considers both medicines to avoid and preferred therapeutic options; supports treatment selection rather than only risk detection	Requires disease-specific clinical interpretation and may not cover every clinical situation	Particularly relevant to clinical prescribing optimisation in older adults with multimorbidity
Renal dose-adjustment guidance [12,90]	Adjustment of medicine selection and dosing according to renal function	Drug accumulation, toxicity, inappropriate dosing in chronic kidney disease	Essential for preventing accumulation and toxicity from renally cleared medicines	Requires current renal-function data; acute changes may make estimates unreliable	Applicable across hospital, primary-care and pharmacy settings when renal function is available
Drug–drug and drug–disease interaction screening [13,37]	Detection of potentially harmful medicine combinations or medicines inappropriate for particular diseases	Bleeding, sedation, serotonin toxicity, QT prolongation, hyperkalaemia, renal injury, cognitive worsening, falls	Highly relevant to multimorbidity and polypharmacy; can identify important safety signals rapidly	May generate excessive or low-value alerts; clinical severity and actionability vary	Useful across electronic prescribing, pharmacy and medication-review systems when followed by clinical assessment
Anticholinergic and sedative burden scales [51,52]	Quantification of cumulative exposure to medicines with anticholinergic or sedative properties	Delirium, cognitive impairment, falls, functional decline, sedation	Captures cumulative medication burden that may not be apparent when individual drugs are considered separately	Different scales classify medicines differently and no universal threshold is established	Useful as an adjunct to medication review, particularly in patients with cognitive impairment, falls or functional decline

Note: the tools are complementary and should be interpreted within the clinical context rather than used as automatic decision rules. Abbreviations: AGS, American Geriatrics Society; EU(7)-PIM, European Union (7) list of Potentially Inappropriate Medications; FORTA, Fit fOR The Aged; GheOP^3^S, Ghent Older People’s Prescriptions Community Pharmacy Screening; MAI, Medication Appropriateness Index; PIPC, Potentially Inappropriate Prescribing Cascades; START, Screening Tool to Alert to Right Treatment; STOPP, Screening Tool of Older Persons’ Prescriptions.

**Table 3:** Belgium and Poland as illustrative European implementation contexts for medication optimisation.

Dimension	Belgium	Poland
Current strengths	Community-pharmacy screening tools (GheOP^3^S), multidisciplinary initiatives such as OptiMEDs, and reimbursed medication review for eligible patients [21,24,99,100].	Large-scale National Health Fund prescribing and claims data, complemented by population-based geriatric evidence and pharmaceutical care [7,8,102].
Clinical implementation focus	Pharmacist-led medication review (type 2A) and collaboration between community pharmacists, general practitioners and patients.	Translation of population-level medication-risk signals into patient-level review involving primary care, geriatrics, pharmacy, nursing, patients, and caregivers.
Key limitation	Fragmented data and variable access to diagnoses, laboratory values, frailty status, treatment goals, and patient preferences [99,100].	Administrative/reimbursement data may not capture over-the-counter medicines, non-reimbursed medicines, or dietary supplements.
Opportunity	Better integration of dispensing, clinical, laboratory, and geriatric data to support risk prioritisation and shared decision-making.	Link national pharmacoepidemiological capacity to clinically actionable medication review and longitudinal pharmacovigilance.

## Data Availability

No new data were created or analyzed in this study. Data sharing is not applicable to this article.

## Abbreviations

The following abbreviations are used in this manuscript:

ADR	Adverse drug reaction
ADE	Adverse drug event
AGS	American Geriatrics Society
AI	Artificial intelligence
CGA	Comprehensive geriatric assessment
DDI	Drug–drug interaction
EU(7)-PIM	European Union (7 countries) Potentially Inappropriate Medication list
FAERS	FDA Adverse Event Reporting System
FORTA	Fit fOR The Aged
GheOP^3^S	Ghent Older People’s Prescriptions Community Pharmacy Screening tool
iSIMPATHY	Implementing Stimulating Innovation in the Management of Polypharmacy and Adherence Through the Years
MAI	Medication Appropriateness Index
NSAID	Non-steroidal anti-inflammatory drug
PCNE	Pharmaceutical Care Network Europe
PIM	Potentially inappropriate medication
PIPC	Potentially Inappropriate Prescribing Cascades
SIMPATHY	Stimulating Innovation Management of Polypharmacy and Adherence in the Elderly
STOPP	Screening Tool of Older Persons’ Prescriptions
START	Screening Tool to Alert to Right Treatment
